# The Case for Community Self-Governance on Access and Benefit Sharing of Digital Sequence Information

**DOI:** 10.1093/biosci/biac019

**Published:** 2022-03-16

**Authors:** Rebecca A Adler Miserendino, Rachel Sarah Meyer, Breda M Zimkus, John Bates, Luciana Silvestri, Crispin Taylor, Tami Blumenfield, Megha Srigyan, Jyotsna L Pandey

**Affiliations:** Lewis Burke Associates, Washington, DC, United States; University of California Santa Cruz, Santa Cruz, California, United States; Harvard University, Cambridge, Massachusetts, in the United States; The Field Museum, Chicago, Illinois, United States; Instituto de Ciencias Humanas, Sociales y Ambientales of the Consejo Nacional de Investigaciones Científicas y Técnicas, Mendoza, Argentina; American Society of Plant Biologists, Rockville, Maryland, United States; Yunnan University, People's Republic of China, and with the Department of Anthropology, University of New Mexico, United States; University of California Santa Cruz, Santa Cruz, California, United States; American Institute of Biological Sciences, Herndon, Virginia, United States

*Digital sequence information* (DSI), a placeholder term commonly understood to refer to information related to genetic sequences stored in a digital format, has become a foundational component to biological research and its applications, including biodiversity conservation and biotechnological innovation. DSI results from the physical access to and use of genetic resources, which falls under the purview of the Convention on Biological Diversity (CBD) and the Nagoya Protocol on Access to Genetic Resources and the Fair and Equitable Sharing of Benefits Arising from their Utilization (NP). The CBD and the NP are legal frameworks governing access to genetic resources and the fair and equitable sharing of benefits arising from their use, a mechanism widely known as access and benefit sharing (ABS). ABS should curb biodiversity loss by increasing incentives for conservation and sustainable biodiversity use. Despite good intentions, a number of national regimes adopted in pursuance of the CBD and NP have created complex, ineffective frameworks that exacerbate the risk of counterproductive effects for biodiversity conservation and sustainable use (Friso et al. 2020).

The debate on DSI focuses on what DSI includes, whether it is covered by the CBD or the NP and the possible implications of its inclusion or exclusion from these agreements. The CBD and NP parties agreed on a science- and policy-based process to debate the treatment of DSI. This process entailed the submission of views and information by parties, other governments, indigenous and local communities, and relevant organizations and stakeholders; the commissioning of technical studies; and the establishment of the Ad Hoc Technical Expert Group (AHTEG) on DSI. The outcomes of the AHTEG were examined by the Open-Ended Working Group on the Post-2020 Global Biodiversity Framework (WG2020), whose recommendations will, in turn, be considered by the CBD Conference of the Parties at its fifteenth conference in Kunming, China, anticipated in summer of 2022. Interim discussions on the issue are taking place at the third meeting of the WG2020 from 14 to 29 March 2022 in Geneva, Switzerland.

To contribute perspectives from the scientific community, the American Institute for Biological Sciences and an informal group of researchers known as the USA Nagoya Protocol Action Group ran an international workshop series called “How does sharing genetic sequence data impact biodiversity science and conservation?” in the fall of 2021 (https://learnnagoya.com/workshop-series/). During this virtual series, funded by the National Science Foundation and conducted in partnership with 18 scientific societies with US and international membership, over 500 participants from 58 countries discussed how DSI is currently managed and what challenges may emerge from explicitly including or excluding DSI under the CBD or the NP. Specifically, we discussed how the scientific community can enhance ABS and conservation in daily practice and in ongoing international discussions. The workshops were focused on experiences of and case studies from archeologists, anthropologists, botanists, biotechnologists, ecologists, microbiologists, plant biologists, virologists, and zoologists. The discussions highlighted that open access to and free circulation of DSI has been the norm within the scientific communities from both developed and developing countries. The participants recognized that benefits arising from the use of genetic resources, including data and knowledge, should be shared in a fair and equitable way, but they also expressed concerns that restrictive regulation of DSI or limits on the open sharing of DSI could further complicate scientific research and innovation (see supplemental material). In the present article, we propose recommendations from the workshops that can contribute to the upcoming discussion on DSI during the WG2020 meeting and beyond.

## Differentiate between commercial and noncommercial use of DSI

Recognizing the value of DSI to support research that addresses global challenges, including biodiversity loss, food security, and global health, the workshop participants emphasized the need to ensure that there is open access to DSI for scientific research. If the ABS frameworks are extended to explicitly address DSI, they should distinguish between the commercial and noncommercial use of DSI. Mechanisms to differentiate between commercial and noncommercial use need to be identified and discussed among CBD and NP negotiators with careful consideration to implementation challenges. Furthermore, the scientific community often needs to respond in real time to global crises, such as preventing the spread of invasive species or vector-borne diseases that can have widespread negative ecological and economic impacts. Whatever policy is agreed to should enable the open sharing of DSI for scientific research across international borders without delay to ensure research can address critical needs.

***Recommendation 1:*** Urge the continued exploration of policy options that differentiate between commercial and noncommercial use of DSI, which can be practically implemented and to enable the open sharing of DSI across international borders without delay.

## Improve data sharing standards through engagement with public data aggregators and DSI users

The workshop series highlighted that scientists who use DSI often expend significant effort to openly share data, through publication and archiving of sequences for open access via public databases such as GenBank and through other initiatives intended to enhance access to genetic information. We recommend not only to raise the visibility of and investments in data aggregators but to request that they curate benefit sharing and permission information to conform with the NP. By making this data available, the scientific and source communities can verify compliance with benefit-­sharing commitments. Implementing this will require coordination across many independent stakeholders who fall outside of the jurisdiction of the CBD.

***Recommendation 2:*** Establish an open forum for major public DSI databases, data aggregators, and users of DSI to explore mechanisms to improve tracking of provenance data and links among DSI, traditional knowledge, and specimens.

## Enable the scientific community to develop best practice guidelines for international collaboration for research using DSI

The scientific community is well positioned to identify opportunities to improve benefit sharing and to develop pragmatic, implementable practices for international collaboration, which may vary by subdiscipline. International representatives of different scientific disciplines and professional societies can be convened to develop guidelines for the responsible use of biodiversity information and its implementations, which involves user awareness about data policies, sample origins, intended use, reporting performed research, recording publications that use repository-hosted data, and encouraging compliance with regulations. There are multiple examples of long-term international research programs that develop communities of practice and mobilize benefit sharing as a part of broadening opportunities for capacity building, engagement, and true intellectual collaboration. Examples include practices for DSI sharing and use created by the WAVE Center of Excellence in Côte d'Ivoire (Bakelana et al. 2019) and the Earth BioGenome Project–Columbia partnership (Huddart et al. 2022).

Inviting members of the scientific community to develop best practices for international collaboration on biodiversity research would be consistent with article 20 of the NP, which establishes that parties “shall encourage, as appropriate, the development, update and use of voluntary codes of conduct, guidelines, and best practices or standards in relation to access and benefit-sharing” and “consider the adoption of specific codes of conduct, guidelines, and best practices or standards.” Engaging with the scientific community to inform these guidelines will increase community buy-in and provide assurances that recommendations will be implementable.

***Recommendation 3:*** Establish a forum under the auspices of the CBD to host international scientists and other users of DSI to develop international best practices for cross-border collaboration.

## Enhance institutional-level support for NP compliance, data sharing, and delivery of benefits

The workshop participants expressed that complying with the NP, even without a mandate to expand its scope to include DSI, has delayed research progress and increased the burden and cost of collaboration. Their institutions provide little to no administrative assistance in navigating NP-related legislation in each country, placing the burden of compliance on the researcher alone, without centralization. Furthermore, many NP parties continue to have inadequate or unclear ABS national regimes, which exacerbates uncertainties about how to share genetic resources across international borders and about personal and professional liabilities associated with lack of compliance.

To address these ongoing and anticipated challenges, governments should provide funding and administrative support to both scientists and research-and-collections-focused institutions to enable compliance with ABS requirements. This could include support for development of legal agreements, permits, material transfer agreements, data sharing, and communication with NP focal points, as appropriate. Without financial support, many scientists and institutions may be disincentivized from pursuing international collaboration, publication, and data sharing.

***Recommendation 4:*** First, urge parties to engage with their domestic science funding agencies to provide institutional-level support to develop domestic infrastructure to support NP compliance. Second, encourage nonparties to voluntarily follow the guidance in the first part of this recommendation, recognizing that scientists from nonparty countries must also comply with the legal frameworks established by NP parties to conduct international research and data sharing.

***Recommendation 5:*** Request CBD Secretariat to develop outreach materials to ensure that institutions and scientists understand their obligations pertaining to the access and benefit sharing of physical specimens and to DSI, as appropriate.

## Increase support for capacity building, collaboration platforms, and infrastructure to enhance biodiversity conservation and resource sharing

The workshop speakers, including those from South America and Africa, emphasized that many biodiversity-rich countries lack capacity, training, funding, and resources to conduct scientific research. In addition, many do not have the infrastructure to archive, publish, or otherwise share DSI or to sustainably house biological specimens. This heightens the importance of international collaborations to maximize nonmonetary benefit sharing to fulfill these functions. If the ABS required under the NP are extended to explicitly include DSI, the delivery of capacity building and training could become further complicated as researchers struggle to understand how to comply with new requirements. For this reason, an increased investment in programs and infrastructure to enhance international collaboration is warranted.

Based on the workshop discussions, two types of programs are especially needed, in addition to increasing support of biodiversity-focused research programs and DSI curation efforts:

Biorepositories are increasingly important facilitators between users and providers, as well as education centers. Biorepositories often provide biological specimens for research and sequencing and also collect and retain user data. Biorepositories are also ABS instruments because they maintain and safeguard genetic diversity. The workshop participants proposed that investments should be directed to maintain and modernize existing, decentralized biorepositories and should support virtual national and international networks to connect distributed biorepositories and researchers. Examples include the virtual community of practice model, Museums and Emerging Pathogens in the Americas (Colella et al. 2021), and the community-derived Extended Specimen Network vision, which would link specimens with their genetic, phenotypic, geographical, and environmental data in alignment with FAIR data principles (for findability, accessibility, interoperability, and reuse; Thiers et al. 2021).

International platforms that support collaborations between scientists in developed countries and those in low and middle-income countries are needed. There are several examples of programs that should be expanded, noting that current support is insufficient to meet demand. The Partnerships for Enhanced Engagement in Research Program, supported by the US Agency for International Development's Higher Education Solutions Network, provides a platform for scientists in low and middle-income countries to partner with US scientists who have received competitive, merit reviewed funding from nine federal research agencies. Similar programs include the Japan Science and Technology Agency, UK Research and Innovation, and the Philippines Department of Science and Technology Science, Technology and Action's Nexus for Development Program, and the African Union and European Union sponsored African Research Initiative for Scientific Excellence Pilot Programme.

***Recommendation 6:*** Recognizing the exemplary practices of growing ABS around DSI in the collections community, direct parties to increase resources to support the development, operations and maintenance of biorepositories, and invite nonparties to voluntarily contribute to these efforts.

***Recommendation 7:*** Encourage parties and nonparties to enhance support of bilateral and multilateral capacity building, collaboration, training and educational exchange programs.

## Encourage states not party to the CBD to reconsider ratification

The conversations about how existing ABS mechanisms can be extended to DSI emphasize the importance for all countries, including the United States, to actively engage in the policy debates occurring in the context of important multilateral environmental agreements, including the CBD. The absence of US negotiators from the policymaking process marginalizes the US scientific community, as well as those in both developed and developing countries who benefit from scientific collaboration and innovation with US scientists.

***Recommendation 8:*** Encourage states not party to the CBD to ratify the agreement as soon as practicable.

## Conclusions

As policy options are considered for how to address DSI in the context of existing ABS mechanisms, including the NP, the implementation framework for each option must be reviewed to assess potential impacts on future technological developments and biodiversity conservation. Policies must preserve open access to DSI for noncommercial intended research, enable international collaboration, be practical, efficient and cost effective to implement, ensure legal certainty, and account for both monetary and nonmonetary benefits; see Scholz et al. (2022) for a recently proposed multilateral mechanism that decouples DSI access from benefit-sharing and addresses these concerns. The scientific community recognizes that the responsible, open availability of DSI democratizes biological research, biodiversity conservation, and innovation. As was highlighted in our international workshop series, the scientific community continues to improve its practices to broaden capacity and enhance intellectual collaboration as a part of benefit sharing with international counterparts. To optimize the effectiveness of ABS frameworks for DSI, policymakers and negotiators must create opportunities to engage with research stakeholders, including DSI users, public biorepositories, data aggregators, science funding agencies, and international scientific societies and enhance support for international biodiversity-focused research and NP compliance.

## Supplementary Material

biac019_Supplemental_File

## Author Biographical

Rebecca A. Adler Miserendino is affiliated with Lewis Burke Associates, in Washington, DC, in the United States (https://orcid.org/0000-0002-1792-4520). Rachel Sarah Meyer is affiliated with the Department of Ecology and Evolutionary Biology at the University of California Santa Cruz, in Santa Cruz, California, in the United States (https://orcid.org/0000-0002-4907-5797). Breda M. Zimkus is affiliated with the Museum of Comparative Zoology at Harvard University, in Cambridge, Massachusetts, in the United States (https://orcid.org/0000-0002-8973-526X). John Bates is affiliated with the Negaunee Integrative Research Center, at The Field Museum, in Chicago, Illinois, in the United States (https://orcid.org/0000-0002-5809-5941). Luciana Silvestri is affiliated with the Instituto de Ciencias Humanas, Sociales y Ambientales of the Consejo Nacional de Investigaciones Científicas y Técnicas, in Mendoza, in Argentina (https://orcid.org/0000-0002-3222-2068). Crispin Taylor is affiliated with the American Society of Plant Biologists, in Rockville, Maryland, in the United States (https://orcid.org/0000-0002-4669-3215). Tami Blumenfield is affiliated with the School of Ethnology and Sociology at Yunnan University, in the People's Republic of China, and with the Department of Anthropology at the University of New Mexico, in the United States (https://orcid.org/0000-0001-5833-0123). Megha Srigyan is affiliated with the Department of Ecology and Evolutionary Biology at the University of California Santa Cruz, in Santa Cruz, California, in the United States (https://orcid.org/0000-0002-8705-8648). Jyotsna L. Pandey is affiliated with the American Institute of Biological Sciences, in Herndon, Virginia, in the United States (https://orcid.org/0000-0001-8173-0424).
