# Supplementary material for: The Case for Community Self-Governance on Access and Benefit Sharing of Digital Sequence Information
Source: Bioscience. 2022 Mar 16;72(5):405–8. doi: 10.1093/biosci/biac019 (PMC9113315; doi:10.1093/biosci/biac019)
Supplement: biac019_Supplemental_File [file biac019_supplemental_file.pdf]

International Workshop Series: How does sharing genetic sequence data impact biodiversity science and conservation?

Final Report
March 7, 2022

Tami Blumenfield, Rachel Sarah Meyer, Rebecca A. Adler Miserendino, Breda M. Zimkus, Megha Srigyan, John Bates, Crispin Taylor, and Jyotsna L. Pandey

This material is based upon work supported by the National Science Foundation under Grant Number 2136950.

TABLE OF CONTENTS

TABLE OF CONTENTS 2

Background & Workshop Series Rationale 4

Synopsis of Workshops 5

Key Themes and Conversations 7

Workshop Overviews 10

- Workshop I: Emerging Pathogen Research Across the Americas 10
  - Discussion 12
  - Recommendations 13
- Workshop II: The role of international collaborations in resolving viral diseases of cassava in Africa 14
  - Discussion 15
  - Recommendations 15
- Workshop III: Enabling Large-Scale Ecological Research with Open Genetic Data 16
  - Discussion 17
  - Recommendations 17
- Workshop IV: More than Potatoes: Collaboration for Collecting and Building the Tree of Life 18
  - Discussion 19
  - Recommendations 20
- Workshop V: International Genomic Research Coordination to Preserve Big Cats 21
  - Discussion 22
  - Recommendations 22
- Workshop VI: Digging into the Bioethics of Studying Humans, from Culture to the Genome 23
  - Discussions 25
  - Recommendations 25

Overall Recommendations 26

Conclusion 27

Important Links 28

References Cited 28

Glossary 30

Workshop Steering Committee **31**

Authors of the Report **31**

1. Background & Workshop Series Rationale

Digital sequence information (DSI), a placeholder term commonly understood to refer to information related to genetic sequences (DNA, RNA, protein) stored in a digital format, has become a foundational component to biological research and its applications, including biodiversity conservation and biotechnological innovation. DSI illuminates the genetic underpinnings of morphological, physiological, behavioral, and functional traits that are the seed corn for innovations to protect and conserve biodiversity in response to various natural and anthropogenic threats.

The period between 2011-2020 was declared "the United Nations Decade on Biodiversity." As a part of this campaign, parties to the United Nations Convention on Biological Diversity (CBD) established the Aichi Biodiversity Targets to drive action towards agreed upon biodiversity conservation goals. In addition, the Nagoya Protocol on Access to Genetic Resources and the Fair and Equitable Sharing of Benefits Arising from their Utilization (NP) entered into force in October of 2014. The CBD and the NP are legal frameworks governing access to genetic resources and the fair and equitable sharing of benefits arising from their utilization, a mechanism widely known as 'Access and Benefit Sharing' (ABS). ABS aims to curb biodiversity loss by increasing incentives for conservation and sustainable biodiversity use. Despite good intentions, a number of national regimes adopted in pursuance of the CBD and NP have created complex, ineffective frameworks that exacerbate the risk of counterproductive effects for biodiversity conservation and sustainable use (Friso et al., 2020). Major policy disagreements and implementation challenges, coupled with the sheer complexity of addressing these global challenges, have left the world short of achieving many of the Aichi Biodiversity Targets. Furthermore, the NP is being implemented without strong global support (130 nations have ratified it, 69 nations, including the United States have not; absch.cbd.int). Very few among the global research and collections communities have the understanding or capacity to comply with aspects of the NP. Many of the disagreements and confusion are about the boundaries of 'what is in the purview' of the NP.

A major debate that has elevated international tensions and concerns among the international scientific community is the debate of whether DSI—which is currently shared as open data (non-commercial) for global benefit—should be regulated under existing ABS frameworks. The debate focuses on what DSI includes, whether it is covered by the CBD and/or the NP, and the possible implications of its inclusion or exclusion from these agreements. While many parties interpret the NP as only applying to *physical* biological specimens, other parties have argued that it should be extended to include DSI as well. Ultimately, DSI results from the physical access to and utilization of genetic resources that fall under the purview of the CBD and NP.

In 2018, CBD and NP parties agreed on a science and policy-based process to debate the treatment of DSI (CBD 14/20, 2018; NP 3/12, 2018). This process entailed the submission of views and information by parties, other Governments, indigenous and local communities, and relevant organizations and stakeholders; the commissioning of technical studies; and the establishment of an Ad Hoc Technical Expert Group (AHTEG) on DSI. Outcomes of the

AHTEG were examined by the Open-ended Working Group on the Post-2020 Global Biodiversity Framework (WG2020) whose recommendations will in turn be considered by the CBD Conference of Parties at its fifteenth conference in Kunming, China, anticipated in summer of 2022. Interim discussions on the issue will take place at the third meeting of the WG2020 from March 14-29, 2022 in Geneva, Switzerland.

While the CBD proceedings are open to registered observers, which includes many academic and science focused organizations, and while there have been several instances in which the CBD has welcomed submissions from stakeholders on various policy options on ABS/DSI, the majority of research scientists remain unaware of ongoing conversations about expanding ABS frameworks to DSI and how this could impact their research programs. Furthermore, there has not been a comprehensive effort to empower members of the global scientific community to generate recommendations for how to promote benefit sharing as a part of international research as it pertains to biodiversity conservation.

To contribute perspectives from the scientific community, the American Institute of Biological Sciences organized an international workshop series called "*How does sharing genetic sequence data impact biodiversity science and conservation?*" in the fall of 2021 (https://learnnagoya.com/workshop-series/). The six-session workshop series hosted on Zoom was funded by the National Science Foundation's Biological Sciences Directorate (DEB-2136950) and conducted in partnership with the USA Nagoya Protocol Action Group (USANPAG) and 18 different scientific professional associations and societies[1]. All partner societies helped to promote the workshops among their membership, while a subset of the societies also co-hosted workshops in the series, with host society leaders and representatives assisting with facilitation. Simply bringing together this diverse group of professional associations, with society representatives providing session introductions that emphasized the importance of the issues discussed to their members, represented a major accomplishment. In part, this served to demonstrate the growing consensus among scientists about the importance of the topics discussed. This report summarizes each workshop event, and provides a synthesis of recommendations generated from workshop discussions with respect to addressing access and benefit sharing as it pertains to DSI.

2. Synopsis of Workshops

The virtual workshop series focused on six separate topics, each featuring a team of transboundary, international scientists who have engaged in international research collaborations. The broad topics around which the sessions were organized included Applied ecology and infectious disease; Crop research and improvement; Macrosystems and international long term ecological research; Phylogenetics, genome evolution, taxonomy; Livestock research and

[1] Partner organizations included the American Anthropological Association, the American Association of Biological Anthropologists, the Botanical Society of America, the American Genetic Association, the American Society for Microbiology, the American Society for Plant Biology, the American Society for Plant Taxonomists, the American Society of Mammalogists, the American Society of Primatologists, the Association for Tropical Biology and Conservation, the Ecological Society of America, the Entomological Society of America, the Global Genome Biodiversity Network, the Global Plant Council, the Helminthological Society of Washington, the Natural Science Collections Alliance, the Phycological Society of America, and the Society for the Preservation of Natural History Collections.

vertebrate genetic rescue; and Anthropology, ethnobiology and paleobiology. The workshop steering committee, with assistance from USANPAG members, worked to identify speakers and recruit host societies for each of the topical workshops.

Speakers represented a broad range of institutions, including museums, zoos, research universities, and nongovernmental organizations, and came from institutional homes in North and South America, Africa, Europe, and Asia. Specifically, the speakers and panelists were from Argentina, Brazil, China, Côte d'Ivoire, Germany, Mexico, the United Kingdom and the United States. The speakers presented a robust set of case studies that illustrated experiences working to collect, analyze and curate DSI.

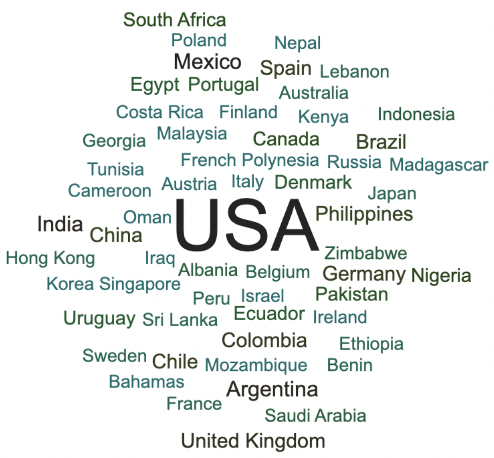

Speakers discussed their efforts and initiatives to comply with existing local, national, and research institution regulations related to their work, many of which represent efforts to comply with international agreements. Presenters also discussed broader ethical and moral issues that transcend these regulations.

Workshop participants who attended the live Zoom sessions came from 58 countries (see word cloud) and totalled over 500 people. Some participants attended multiple workshops. The workshop recordings were used in classrooms, such as UC Santa Cruz's Molecular Ecology course. As of March 2, 2022, the six workshop videos have been watched 420 times.

Each virtual session was introduced and led by Dr. Jyotsna L. Pandey, PI, while Dr. Rachel Meyer, co-PI, provided information about the LearnNagoya.com website and its resources, including the workshop discussion forum established to facilitate community engagement after the workshops. After a general introduction, host society leaders provided brief introductory remarks about why the topics of DSI regulation and access and benefit sharing were important for their membership and introduced the featured speakers. During each workshop, the presentations were kicked off with a brief overview of the policy landscape. In four of the six workshops, steering committee co-chair Dr. Rebecca Adler Miserendino provided context on current debates and discussions of DSI, various related terminologies, the scope of their usage in different contexts and issues around DSI sharing. For the remaining two sessions, the policy presentation was given by Dr. Luciana Silvestri, an environmental lawyer from the Instituto de Ciencias Humanas, Sociales y Ambientales (INCIHUSA) in Argentina. The discussion that followed the featured talks was facilitated by host society representatives with assistance from steering committee members, Dr. John Bates (co-PI), Dr. Breda Zimkus, and Dr. Crispin Taylor.

Workshop participants discussed how DSI is currently managed and what challenges may emerge from explicitly including or excluding DSI under the CBD and/or the NP. Specifically, conversations focused on how the scientific community can enhance ABS and conservation in daily practice and in ongoing international discussions. The workshops showcased the

experiences of and case studies from archeologists, anthropologists, botanists, biotechnologists, ecologists, microbiologists, plant biologists, virologists, and zoologists. Discussions highlighted that open access to and free circulation of DSI has been the norm within the scientific communities from both developed and developing countries. Participants recognized that benefits arising from the use of genetic resources, including data and knowledge, should be shared in a fair and equitable way, but they also expressed concerns that restrictive regulation of DSI or limits on the open sharing of DSI could further complicate scientific research and innovation.

3. Key Themes and Conversations

Several common themes emerged from the six workshop sessions. Participants emphasized that approaches to regulating DSI must **preserve open access and enable international collaboration**. International collaboration and the free flow of scientific information and communication are crucial for scientists to address pressing challenges, such as spread of infectious disease among plants, animals and humans in a timely manner. In fact, open-data requirements of many funding agencies and universities have been broadly adopted by scientists and professional communities (for details, see resources on the FAIR—Findable, Accessible, Interoperable, Reusable—Data Principles for Scientific Data Management and Stewardship, https://www.go-fair.org/fair-principles/; Wilkinson et al., 2016).

Speakers emphasized **the urgent need for DSI-related research for biodiversity conservation**, as well as the need for international coordination. Dr. Oliver Ryder of the San Diego Zoo discussed the significance of numerous international consortia that have emerged to pool resources and data about different taxonomic groups. These include the Vertebrate Genomes Project, Zoonomia, China-based BGI, GIGA, and Genomics for Australian Plants. As Dr. Ryder noted, "The rapid proliferation of DNA sequence information is going to change our understanding of the world." He also emphasized that "expanded efforts in biobanking, especially for viable living cellular resources, are urgently needed to conserve biodiversity and provide options for preventing species extinctions."

Another recurring topic of discussion was the **need to differentiate between commercial and non-commercial uses of DSI**. It was suggested that differentiating between the intended uses could allow research without commercial intentions to proceed without onerous requirements for compliance. However, it was also recognized that boundaries between commercial and noncommercial intended use projects can often be fluid. Research projects originally designed for non-commercial uses can later lead to commercial use. Furthermore, if data are openly shared in alignment with FAIR data principles, then mechanisms would need to be established to track when DSI are utilized for commercial purposes. Recognizing the value of DSI to support research that addresses global challenges, including biodiversity loss, food security, and global health, workshop participants emphasized the need to ensure that there is open access to DSI for scientific research. If ABS frameworks are extended to explicitly address DSI, they should distinguish between the commercial and noncommercial use of DSI. Furthermore, mechanisms to differentiate between commercial and noncommercial use need to be identified and discussed with careful consideration to implementation challenges. Importantly, the scientific community

often needs to respond in real time to global crises, such as preventing the spread of invasive species or vector-borne diseases that can have widespread negative ecological and economic impacts. Ultimately, whatever policy is agreed upon must enable the open sharing of DSI for scientific research across international borders without delay to ensure research can address critical needs in a timely manner.

It was argued that **expected boundaries between "user countries" and "provider countries" are much less clear** than the Nagoya Protocol may have anticipated: significant overlap exists between the top user countries and the top provider countries. As speaker Dr. Jocelyn Colella argued, the idea of binary interpretations of countries as 'Providers' or 'Users' perpetuates "an old model of colonialism" and is "simply inaccurate." Citing a recent research study, she noted that the US, China, and Canada were among the top providers and users of DSI.

Participants discussed the **unique and important role of biorepositories**, which are increasingly important facilitators between 'users' and 'providers' as well as education centers. Biorepositories often provide biological specimens for research and sequencing, and collect and retain user data. Biorepositories are also ABS instruments because they maintain and safeguard genetic diversity. Workshop participants proposed that investments should be directed to maintain and modernize existing, decentralized biorepositories and should support virtual national and international networks to connect distributed biorepositories and researchers. Examples include the virtual community of practice model, Museums and Emerging Pathogens in the Americas (MEPA) (Colella et al., 2021), and the community-derived Extended Specimen Network vision, which would link specimens with their genetic, phenotypic, geographical, and environmental data in alignment with FAIR data principles (Thiers et al., 2021).

Participants highlighted the **importance of inclusive and community-regulated consortia for facilitating long-term collaboration and data sharing**. Consortia policies are developed by communities of practice, and are able to evolve in concert with community bioethics. There are multiple examples of long-term international research programs that develop communities of practice and mobilize benefit sharing as a part of broadening opportunities for capacity building, engagement, and true intellectual collaboration:

1) The WAVE Center of Excellence in Côte d'Ivoire facilitates rapid sharing of DSI and observations of plant pathogens across 14 countries, and provides the WAVE Cube cyberinfrastructure to help users explore datasets, including viral genomes not shared in other global repositories (Bakelana et al., 2019).
2) The Earth BioGenome Project and associated projects, such as Genome 10k, the Vertebrate Genome Project, Zoonomia, and Genomics for Australian Plants, set reference genome standards, negotiate better rates for supplies and sequence data as conglomerates, and help fast-track genomics programs in developing countries. For instance, the Earth BioGenome Project-Columbia partnership (Huddart et al., 2022).
3) The National Ecological Observatory Network and iLTERs allow different communities to develop their own best practices and standards around data sharing.

4) International laboratories such as the ELDORADO LMI in Mexico City provide training, data generation and data stream integration on emerging animal diseases to regional partners in Haiti and Guatemala, where facilities are insufficient.
5) ASIAPAST links DSI with other critical data from culturally connected countries to describe hunter-gatherer and mobile pastoralism practices over millennia. In so doing they make tremendous efforts to organize and preserve biomaterials in underfunded or at-risk repositories, enhancing local infrastructures and disincentivizing specimen hoarding through collaboration.
6) The Planetary Biodiversity Inventories (PBI) *Solanum* Program coordinated field collections and DSI generation and analysis among the globally important *Solanum* genus (that includes tomato, eggplant, potato, etc.) in Africa, South America, the US, UK, and Madagascar, which enabled the discovery and delineation of species for the Flora Brazil 2020. The program helped bring international researchers to institutions that are normally peripheral to funding and collaboration opportunities available in major research centers, including small herbaria and universities in the Amazon.
7) The Global Alliance for Genomics and Health (GA4GH) initiative seeks to bring together a federated team of scientists to improve human reference genomes, after observing decades of global inequity in health sciences because the original human genome was a white male. This NHGRI-funded initiative is designed to release a globally representative human pangenome reference constructed from more than 350 individuals representing highly diverse ethnic backgrounds.

Speakers noted that enabling such consortia to accelerate capacity building at local and regional scales and supporting international programs through multiple grant cycles would be critically important for empowering biodiversity science and conservation.

Finally, participants noted that **the continuum of *equity*, *justice,* and *efficiency* is critical**. Many speakers noted the importance of allocating time in international projects to build capacity within collaborating universities and institutions, and to engage with people from local communities. Workshop speakers, including those from South America and Africa, emphasized that many biodiversity-rich countries lack capacity, training, funding, and resources to conduct scientific research. In addition, many do not have infrastructure to archive, publish, or otherwise share DSI or to sustainably house biological specimens. This heightens the importance of international collaborations to maximize non-monetary benefit sharing to fulfill these functions. Further, speakers noted that many collaborations were already providing non-monetary benefits, regardless of existing benefit-sharing agreements, and argued that researchers should articulate in their grant proposals how the proposed work can benefit the partner country by clearly outlining all non-monetary benefits that would result from it. Participants emphasized the need for research projects to include sufficient time and resources not only for complying with international agreements, but also for supporting capacity building initiatives, such as training for students and researchers in the country where the research is being conducted. Including in-country collaborators on research teams and publications, presenting research results at institutions in the provider country, and learning about and offering feedback on local research projects would all be potential ways to accomplish this.

4. Workshop Overviews

Workshop I: Emerging Pathogen Research Across the Americas
(Watch Recording)
Topic: Applied Ecology and Infectious Disease

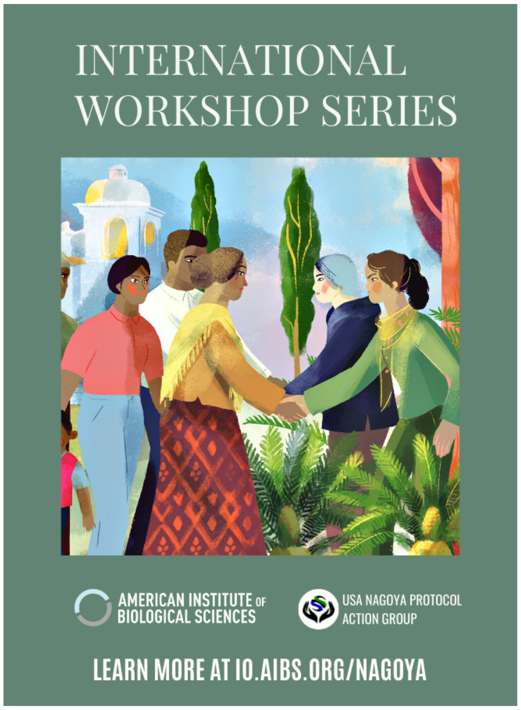

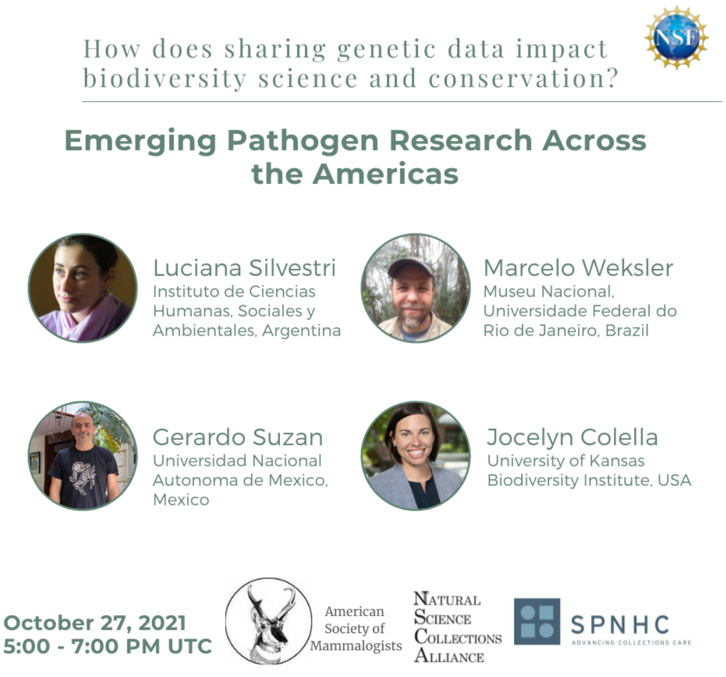

The opening workshop, hosted by the American Society of Mammalogists, the Natural Science Collections Alliance, and the Society for the Preservation of Natural History Collections, was attended by 173 participants from 30 countries representing North America, South America, Asia, Europe, Africa, and Oceania. Sixty percent of attendees reported that they work with biological collections; 60% have active international scientific collaborations; and 73% work with DSI.

The first talk, titled "Understanding the international debate on Digital Sequence Information: What is at stake" was presented by Luciana Silvestri, an environmental law researcher from the Instituto de Ciencias Humanas, Sociales y Ambientales (INCIHUSA), Argentina. Dr. Silvestri introduced the topic of DSI, including terminologies, scope and relevant issues. She discussed how the scientific community can engage in the policy debate around DSI sharing, including through frequent and clear communication of their objectives and seeking to understand providers' position. Dr. Silvestri emphasized the importance of making distinctions between commercial and non-commercial use of DSI, as well as finding ways to utilize such data in responsible ways by ensuring traceability and robust database policies.

The second talk, titled "Navigating Nagoya Protocol's turbulent waters in Brazil: Perspectives from researchers on basic biodiversity science" was presented by Dr. Marcelo Weksler, Full Professor and Curator of Mammals at the Museu Nacional, Universidade Federal do Rio de Janeiro, Brazil. Dr. Weksler is also part of the university-wide working group on NP related issues. The talk was an informative, firsthand account of experiences in conducting basic biodiversity research in Brazil's unique ecosystems, where many species remain unsequenced. Dr. Weksler noted there were substantial regulatory hurdles faced by researchers. In Brazil, genetic resources are considered protected genetic heritage. Regulations prohibit foreigners from accessing specimens unless they collaborate with a Brazilian researcher, provide geo-referencing of sampling locations, complete registration at specific moments in the procurement process, and follow rules governing the publication and commercialization of research. Dr. Weksler detailed the significant shift towards bureaucracy and red tape since NP-related regulatory frameworks were introduced, making basic research very onerous.

The third talk, titled "Ecological approaches for the study of infectious diseases: Examples from Neotropical and Nearctic Mexico" was presented by Dr. Gerardo Suzán, a Doctor of Veterinary Medicine with a Master of Science in Ecology from the National Autonomous University of Mexico (UNAM), and PhD in Biology from The University of New Mexico. Dr. Suzán discussed how anthropogenic activities including unsustainable land use, intensive farming and habitat degradation have modified the wildlife-livestock-human interface, making us susceptible to a wide variety of infectious agents that mutate, adapt, and become resilient, thereby increasing risk of infectious diseases like the current pandemic. Dr. Suzán noted that the Nagoya Protocol could be helpful to regulate genetic resources, enhance traditional knowledge, result in faster pathogen identification, enhance intersectoral collaborations, and importantly, reduce biopiracy. However, this would require significant coordination at the administrative, legislative and policy levels. An example of a promising international collaborative effort is the ELDORADO project, which assesses biodiversity, habitat modifications, and risk of emerging pathogens and disease in the Yucatan, Mexico.

The final talk of the workshop was presented by Dr. Jocelyn Colella, Curator of Mammals at the University of Kansas (KU) Biodiversity Institute, Assistant Professor of Ecology and Evolutionary Biology, and co-founder of 'Museums and Emerging Pathogens in the Americas (MEPA).' Dr. Colella linked colonial histories with contemporary research and data sharing practices and urged recasting of interactions among governments, companies, researchers, and communities within a justice framework. She emphasized the importance of biorepositories as primary infrastructure for emerging pathogen research and the role of museums as potential facilitators between "users" and "providers" of genetic information. She discussed the MEPA initiative (Figure 1), which aims to unite biorepositories with ongoing biomedical and pathogen surveys and serve as a global decentralized pathogen surveillance network (Colella et al., 2021).

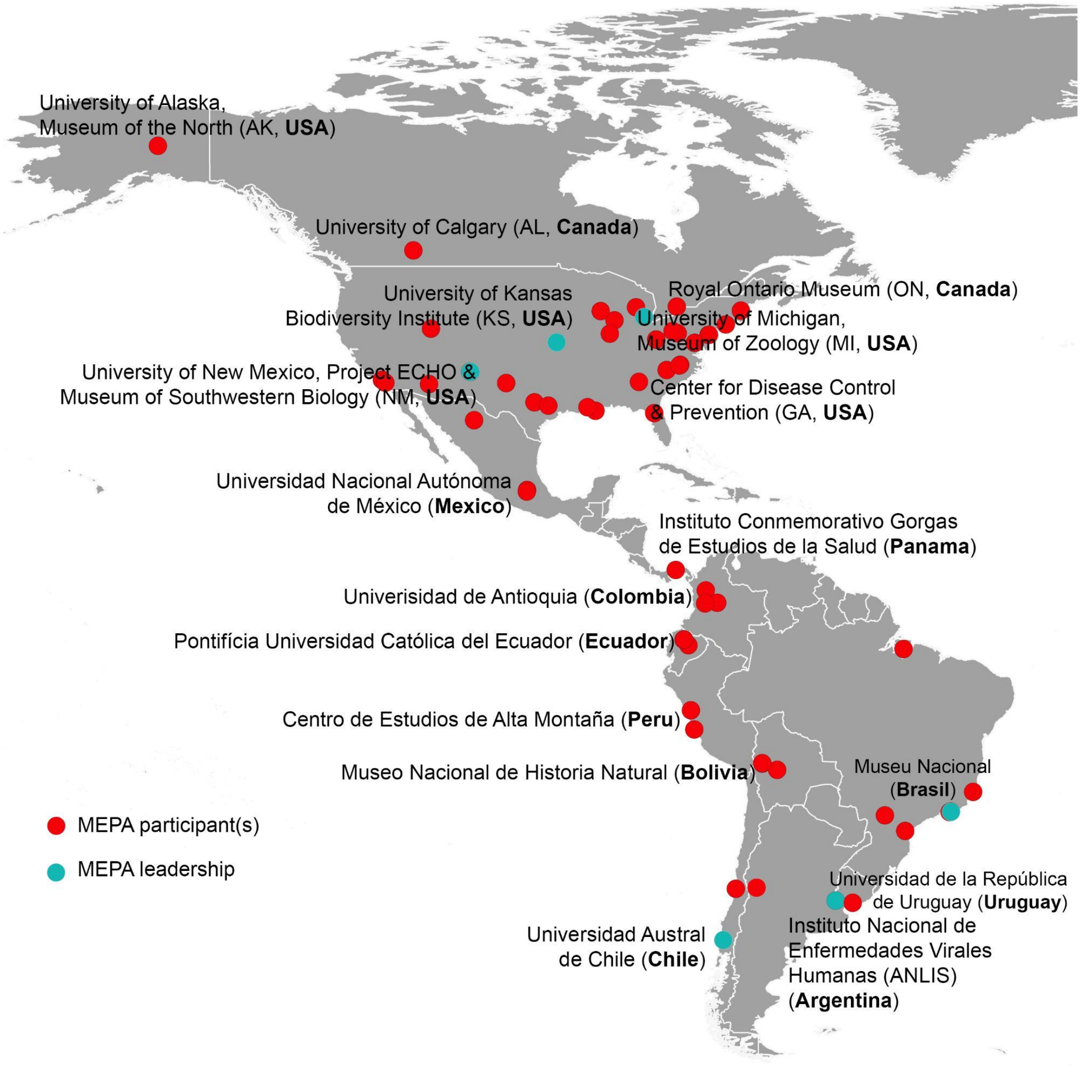

Figure 1. Distribution of institutions participating in Museums and Emerging Pathogens in the Americas (MEPA). At least one participating institution is labeled in red per country, with all leadership institutions in turquoise. (Source: https://hsc.unm.edu/echo/partner-portal/programs/mepa)

Discussion

Panelists delved into several topics during the discussion, including (1) the value of biorepositories and museums as national access points; (2) the importance of connecting or linking organismal collections with their DSI and other metadata, through persistent identifiers (PIDs) and specimen vouchers, especially for pathogen-related genetic information; (3) navigating commercial and non-commercial DSI usage; and (4) penalties for non-compliance with Nagoya Protocol regulations, even for researchers from non-signatory countries.

Recommendations

The following ideas emerged from conversations taking place during the workshop:

1. Focus on non-monetary benefits when considering benefit sharing strategies:
    a. Emphasize and prioritize training aspects while conducting data collection, management and analysis
    b. Provide access to information relevant to conservation and/or sustainable use of biological diversity;
    c. Offer admittance to databases and to ex situ facilities of genetic resources;
    d. Promote collaborative activities and research;
    e. Offer joint ownership of relevant intellectual property rights;
    f. Participate in product development;
    g. Focus research on areas of priority (e.g., health and food security) for host country and local communities;
    h. Consider social recognition;
    i. Use and share appropriate technology; ensure knowledge transfer to the provider of the genetic resources.
2. Repatriate benefits resulting from DSI information, as well as specimens, in a culturally sensitive and appropriate manner that is respectful of the sentiments of the nation or concerned communities. Maintain and rapidly scale up capacity to safely store specimens and DSI. The utmost priority is the specimens and DSI over cultural-political wants. The biodiversity loss from unstable human situations is not recoverable.
3. Inquire about and identify specific needs of local communities while devising benefit sharing strategies. Make sure that these needs are met and adequately represented.
    a. In addition to training, include local students and researchers as authors in joint publications, supply educational resources such as books, make investments in basic educational infrastructure, etc.
4. Share information about Nagoya Protocol focal points and relay experiences from field work in other countries, in order to create a common ground for discussing issues in a particular country
5. Leverage the potential of scientific societies in fostering ABS:
    a. Curate relevant resources around DSI access and sharing to help younger researchers navigate regulatory landscapes
    b. Partake in negotiations around access and sharing of data
    c. Encourage trans-national collaborations by sharing funding for scientific positions that are sponsored by multiple societies, thus easing financial burden on a single institution
    d. Maintain a proactive organization within the society that stresses firmly on researchers being aware of their bigger responsibilities beyond data acquisition/analysis.
6. Advocate for non-monetary benefit sharing.

Workshop II: The role of international collaborations in resolving viral diseases of cassava in Africa
(Watch Recording)
Topic: Crop Research and Improvement

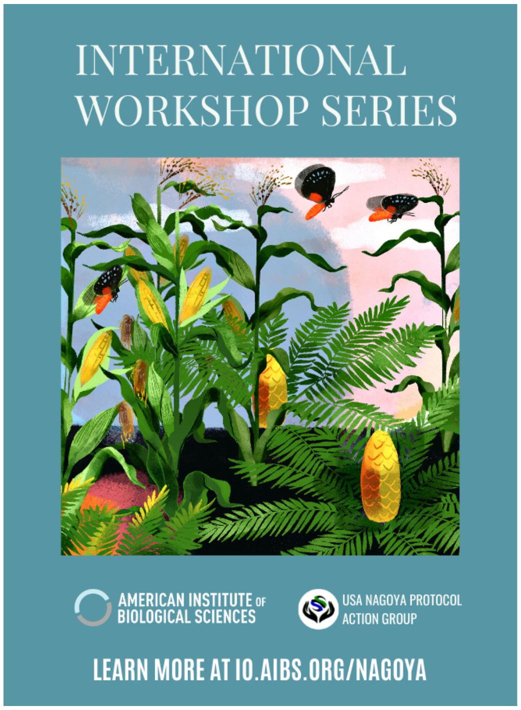

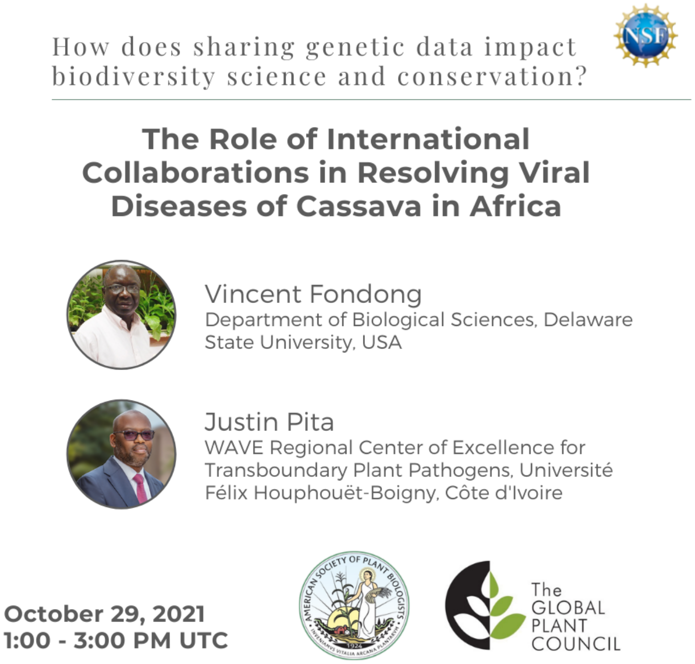

This workshop, hosted by the American Society of Plant Biologists and the Global Plant Council, was attended by 34 participants from 14 countries representing North America, Asia, Europe and Africa. The program started with an overview of the policy landscape by Dr. Rebecca Adler Miserendino, a public health and environmental scientist with over a decade of professional experience in both academic research and international policy. Dr. Adler Miserendino talked about digital sequence information, terminologies, scope and issues, which was followed by talks by guest speakers.

The first talk, "The Role of International Collaborations in Resolving Viral Diseases of Cassava in Africa", was presented by Dr. Vincent N. Fondong, a professor of biotechnology at Delaware State University, who works on the molecular biology of plant viruses and development of virus-resistant plants, with particular focus on Geminiviruses and Potyviruses, the two most devastating plant viruses worldwide. Dr. Fondong outlined how attempts to address the cassava virus crisis have created a situation whereby laboratories in the 'Global North' generate the sequencing data and provide most of the funding, while local scientists who could contribute contextual knowledge about disease and community circumstances are primarily involved in field collection. In-country collaborators are frequently included as co-authors on publications but are not usually involved in experimental design. Finally, not all viral genomic data is shared in public repositories.

The next talk, "Cassava Viral Diseases (CVD) in Africa: Reversing the Tide," was presented by Dr. Justin Pita from the WAVE Regional Center of Excellence for Transboundary Plant Pathogens, Université Félix Houphouët-Boigny, Côte d'Ivoire. Dr. Pita introduced WAVE (Central and West African Virus Epidemiology), an alliance of 14 countries who are trying to reverse the tide of the viral infection in cassava plants. Along with national agricultural agencies, their objective is to increase the productivity and sustainability of tuber crops in Africa through coordinated management of viral disease threats. The WAVE alliance exemplifies a well-coordinated collaboration, where all partners follow the same protocol of data collection; work under clearly established rules about clarity, transparency and fairness; and follow procedures to establish prior informed consent and mutually agreed terms. Partner universities outside Africa have provided technical expertise and assistance, including providing electronic tablets for data collection. Also, the alliance has developed the 'WAVE-cube', a cutting-edge approach to viral disease data storage and management that serves as an early surveillance/warning system for plant disease, using an interactive platform to enable data visualization and disease spread modeling.

Discussion

Aspects discussed included how the WAVE alliance incorporates ABS principles in DSI sharing, whether additional country-specific agreements exist, and how various levels of partnerships with participating countries operate at the government, ministry or agency-level. Issues relating to inequitable funding between the global north vs. global south partner countries and reluctance from funders in allocating large amounts of funds to less established universities were discussed. Speakers also discussed improving data sharing and how benefits generated could be shared with local researchers and collaborators.

Recommendations

The following ideas emerged from conversations taking place during the workshop:

1. Make grants more inclusive of local researchers, including in terms of experimental design and scientific planning.
2. Use grant funding resources to strengthen existing administrative and financial procedures in African institutions. Dr. Fondong gave an example of the International Science Foundation in Stockholm, Sweden that involved creating a Swedish bank account that could be used to transfer money directly to local researchers in Africa. Establishing similar systems could ensure that the local administration does not encroach upon or mismanage the money.
3. Develop capacity of local laboratories and train personnel in African countries.
4. The international community outside the WAVE funding framework could help in the efforts against the spread of cassava viral diseases from East to West Africa by voicing their concerns on international platforms.
5. Make the genomic sequence data more accessible, and design strategies to expand data usage for local researchers beyond their local computers.
6. Leverage local traditional knowledge associated with crop diseases.

Workshop III: Enabling Large-Scale Ecological Research with Open Genetic Data
(Watch Recording)
Topic: Macrosystems and International Long Term Ecological Research Learning Portal

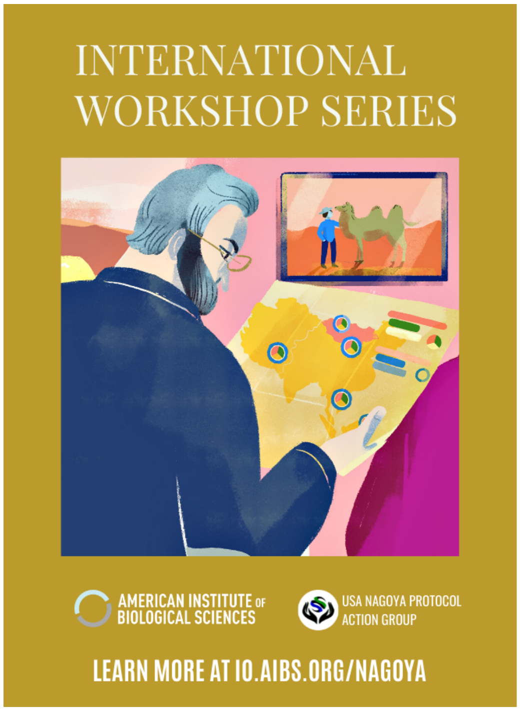

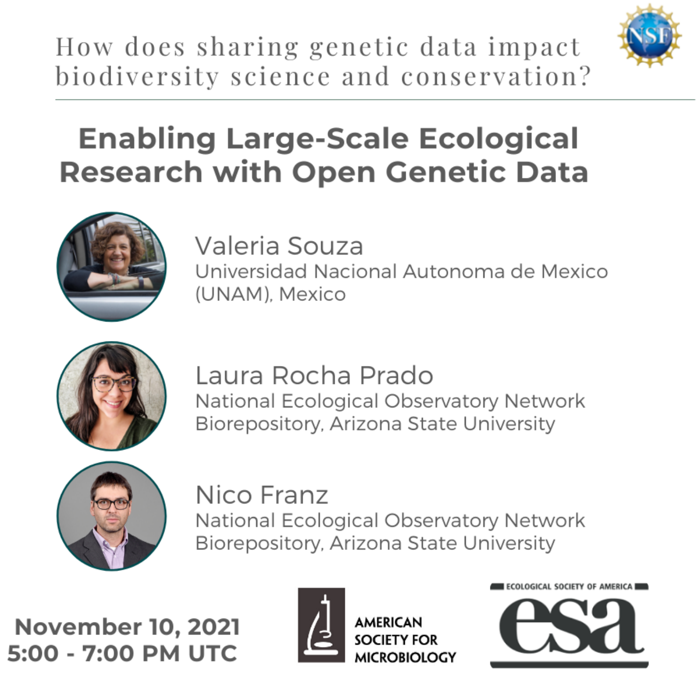

The workshop, hosted by the American Society for Microbiology and the Ecological Society of America, was attended by 59 participants from 20 countries representing North America, South America, Asia, Europe, Africa, and Oceania. The program started with an overview of the policy landscape by Dr. Luciana Silvestri, who discussed the definition of DSI, various related terminologies, the scope of their usage in different contexts and issues around DSI sharing.

The first talk, titled "Capacity Building as a Way to Follow Nagoya: Examples fromMexico and Chile", was presented by Dr. Valeria Souza from the National Autonomous University of Mexico, whose research focuses on understanding the evolutionary, physiological, and ecological processes of microorganisms. Dr. Souza introduced the site of Cuatro Cienegas Basin in Mexico, where microbial diversity is strong but threats from agriculture-related water drainage and habitat destruction hover. Both here and in a second site in Chile's Magallanes region, Dr. Souza has been involved in long-term efforts to instill the value of protecting natural genetic resources. Children from a very young age are exposed to this in art as well as science workshops, and the bioprospecting is conducted by youth of this region. This is the first example of long-term capacity building towards the Nagoya Protocol in Mexico. Meanwhile, multiple ocean currents and numerous migratory species in the Magallanes region offer an important site for gathering genetic resources of this region and studying how these are impacted by climate change.

The next talk, titled 'Fostering Open Biodiversity Data Communities: The NEON Biorepository Example' was presented by Dr. Laura Rocha Prado, Biodiversity Informatician working in the National Ecological Observatory Network (NEON) Biorepository, Arizona State University, USA and Dr. Nico Franz, the Virginia M. Ullman Professor of Ecology and Director of Biocollections in the School of Life Sciences, Arizona State University (ASU). The NEON biorepository is a network of shareable data, envisioning decentralization along with global coordination of biodiversity data, which encourages the emergence and evolution of multiple self-identifying communities of practice that are regionally, taxonomically or institutionally localized. Each community can make decisions about the appropriate manner of data sharing, especially where target species may be rare or endangered and have restrictions on data access.

Discussion

Could creating a microbiology credit/currency similar to the dollarized carbon credit help conserve unique environments? Dr. Souza remarked that this could be useful to help protect endangered stromatolite habitats that are found in extreme environments, not only in Cuatro Cienegas, but also in other similarly fragile areas that capture carbon and could serve as potential resources for future agricultural, medicinal, and antibiotic solutions. Potential applications of block-chain technologies to conservation concerns were also discussed. Defining essential biodiversity variables and scaling biodiversity information temporally and spatially could contribute to forecasting and decision making on national and international policy arenas. Aspects of DSI related to legacy samples were discussed, especially cases where sample origins are unknown. FAIR versus CARE data trade-offs, constraints on fair practices, metagenomic versus whole genomic data usage, related DSI terms, and specific legislations for commercial versus non-commercial use were debated.

Recommendations

The following ideas emerged from conversations taking place during the workshop:

1. Seek improved coordination of governmental agencies in Mexico (and elsewhere) towards protecting water, perhaps through international pressure.
2. Integrate ecological data into global and national public health policy as well as climate change policymaking.
3. Emphasize bottom-up versus top-down policy making, investing into mechanisms that translate data into public policy decisions and expanding long-term monitoring of ecological data over time such as through existing networks in Brazil, USA, and Mexico.
4. Implement systems that require creating partnerships with local researchers, taking advantage of established funding structures and restricting data sharing with private entities before returning benefits to the research community to ensure equitable distribution.
5. Consider developing and delivering educational tutorials like microbiology courses on ribosomal and genomic data analyses to support student training.
6. Consider creating checkpoints that control malicious intent or data usage through patents offices or other existing agencies.
7. Disclose patents and how they were obtained when publishing articles.

8. Define and promote responsible use of biodiversity information and its implementations, which involves user awareness about data policies, sample origins, intended use, reporting performed research, recording publications that utilize repository-hosted data and encouraging compliance with regulations.

Workshop IV: More than Potatoes: Collaboration for Collecting and Building the Tree of Life
(Watch Recording)
Topic: Phylogenetics, Genome Evolution, Taxonomy

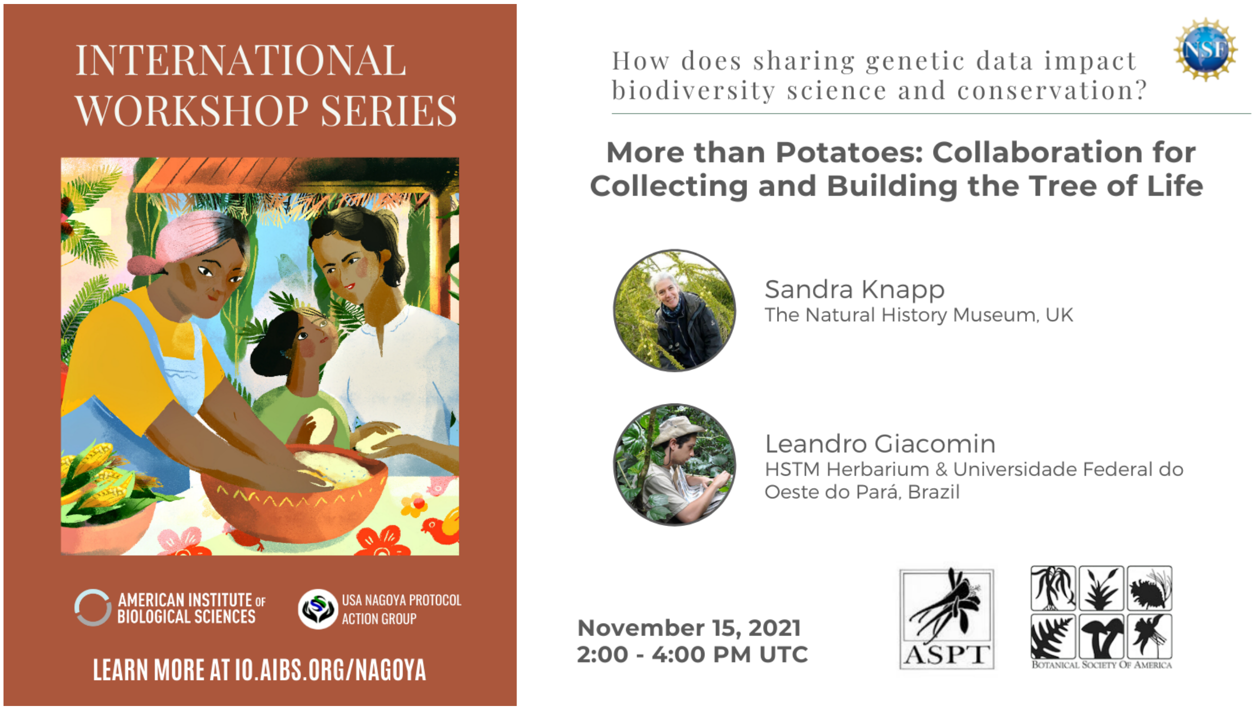

This workshop, hosted by the American Society of Plant Taxonomists and the Botanical Society of America, was attended by 121 participants from 31 countries, representing North America, South America, Asia, Europe, Africa, and Oceania. It provided a space for sharing ideas and questions about phylogenetic research on species in the *Solanum* genera of plants, including the potato, tomato and eggplant, which are important food crops. The workshop began with an introduction by Dr. Rebecca Adler Miserendino, who discussed the definition of DSI, various related terminologies, the scope of their usage in different contexts, and issues around DSI sharing.

Co-presenters Dr. Sandra Knapp, from The Natural History Museum, UK and Leandro Giacomin, from the HSTM Herbarium & Universidade Federal do Oeste do Pará, Brazil, discussed their research and initiatives related to *Solanum*, in a talk titled "More than Potatoes: Collecting and Building the Tree of Life". One-fourth of *Solanum* (Figure 2) occur in Brazil, yet this group remains taxonomically poorly resolved. The genus shows huge

vegetative variation yet has a conserved flower structure, which is a taxonomic paradox and forms the basis for taxonomic research into this genera. The Solanum programme aims to create an open community dataset that supports collaborative projects. Datasets include vouchered data and extended digital specimens that include geographical coordinates, biological sequences, specimens as well as information about their evolutionary framework, all of which are important for collaborative projects.

Flora Brazil 2020, an initiative that aims to compile and describe all species occurring in the country, has achieved monographs for nearly all genera except *Solanum*. Despite having an advanced system of biodiversity data organization in Brazil, huge gaps have been identified in making the data more accessible to a wider range of people instead of being concentrated in main cities and universities, where information is hosted on platforms such as GBIF, CRIA and REFLORA. Challenges around the project include the lack of funding to fill the existing gaps in primary data, distinguishing between commercial and non-commercial uses of data and notably, the changing nature of sequence data through time and coordinating different international tracking systems to link diverse data types.

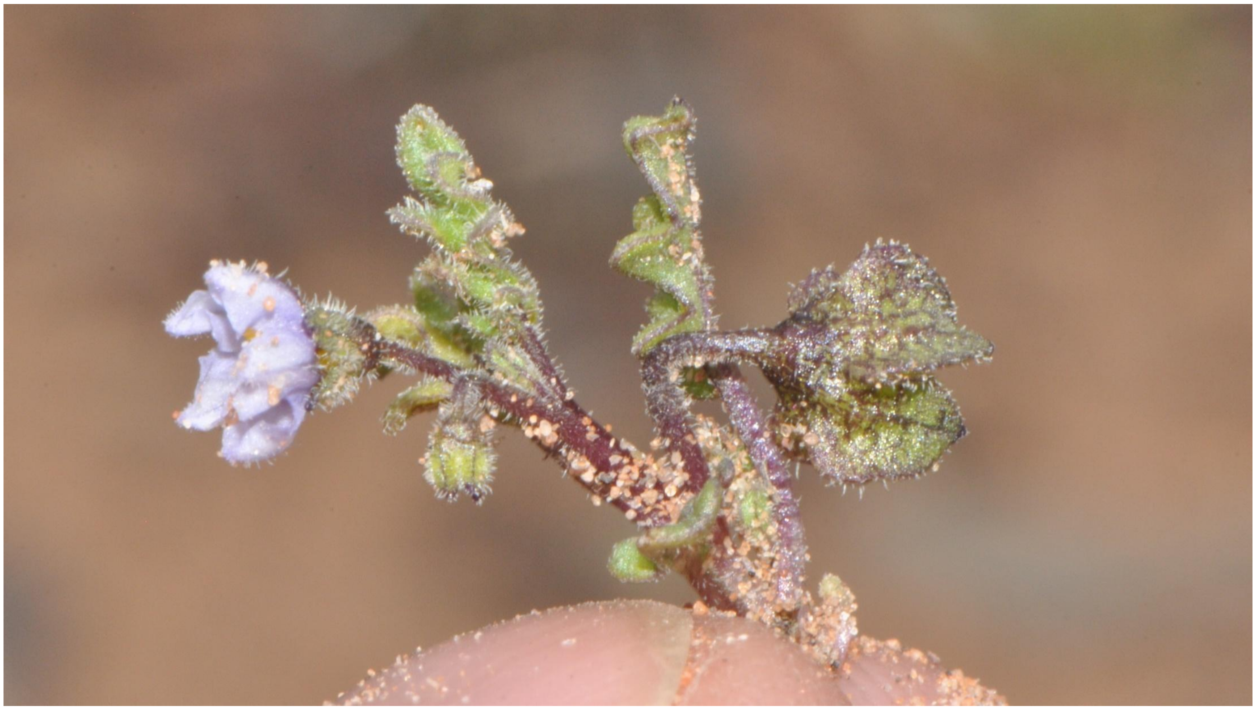

Figure 2: *Solanum weddellii* Phil. (Source: speaker slides)

Discussion

The discussion began with questions about respective permits required for US-based taxonomists and plant breeders both in the US and Brazil. In general, this is a complicated area due to different end-products intended by taxonomists (i.e., herbaria specimens) versus plant breeders (seeds) as well as differing rules at each step of breeding programmes, e.g., from the pre-breeding step to actual breeding, and subsequent agreements with large corporations.

Researchers must take steps to avoid biopiracy by reading the fine print of these regulations and making efforts to understand commercial versus non-commercial usage, especially as breeders continue to expand their interests to wild relatives of crops other than tomatoes, potatoes and aubergines. There are differences between the Nagoya Protocol, which does not have a multilateral mechanism for monetized benefit sharing but might allow both monetary and non-monetary benefits to be negotiated as Mutually Agreed Terms, versus the International Treaty for Plant Genetic Resources, which has established monetized benefit sharing. Acknowledgement of the efforts of national agencies towards conservation of a target crop is also a good step towards avoiding helicopter science and part of ensuring justice and sovereignty for the provider country. Points were raised about FAIR data sharing principles while respecting indigenous knowledge and preventing unconsented data. Since accessing permits to obtain traditional knowledge is very time-consuming, researchers often prefer to conduct taxonomic or evolutionary research, but need to carefully navigate bureaucratic procedures to acquire prior informed consent to access traditional knowledge. This is especially important as many indigenous communities are subject to environmental degradation and have fragile sustenance, thus sharing benefits generated is paramount for fairness.

Next, given the complicated regulations around establishing agreements with various countries, the idea of an additional layer of coordinating group that could triage between different partners was discussed to help streamline legislations. Possibilities to streamline the entire trajectory from sample collection to generating data were discussed, such as ensuring clarity about the intended usage of the downstream product, standardizing permit processing across countries through international treaties and understanding related challenges such as climate change.

Recommendations

The following ideas emerged from conversations taking place during the workshop:

1. Prioritize justice over efficiency at various steps in the research, data analysis, and data sharing and publication processes, even if it takes more time to generate scientific outputs. Integrate capacity building goals explicitly in the design and implementation phases of a project.
2. Help train and support the next generation of scientists, improving local science literacy, data quality, and the possibility of using sequence data in other projects.
3. Make data more accessible to a wider range of people instead of remaining concentrated in main cities and universities by training more people in diverse settings, upgrading database systems, and allocating more funding to local researchers.
4. Use agreements like the Nagoya Protocol to support mutual exchange of data through portals such as GBIF, CRIA, and REFLORA to foster transparency and collaboration.
5. Improve funding to fill the existing gaps in primary data.
6. Distinguish between commercial and non-commercial uses of data.
7. Given the changing nature of sequence data through time, coordinate different international tracking systems to link diverse data types.
8. Focus on trust-building by investing time in developing relationships to facilitate meaningful collaborations.

9. Leverage local herbaria, and enhance existing herbaria networks, for archival and monitoring purposes.
10. Leverage international scientific societies to help researchers navigate permits, and enhance inclusivity for researchers from smaller institutions in forming trans-national collaborations.

Workshop V: International Genomic Research Coordination to Preserve Big Cats
(Watch Recording)
Topic: Livestock Research, Vertebrate Genetic Rescue

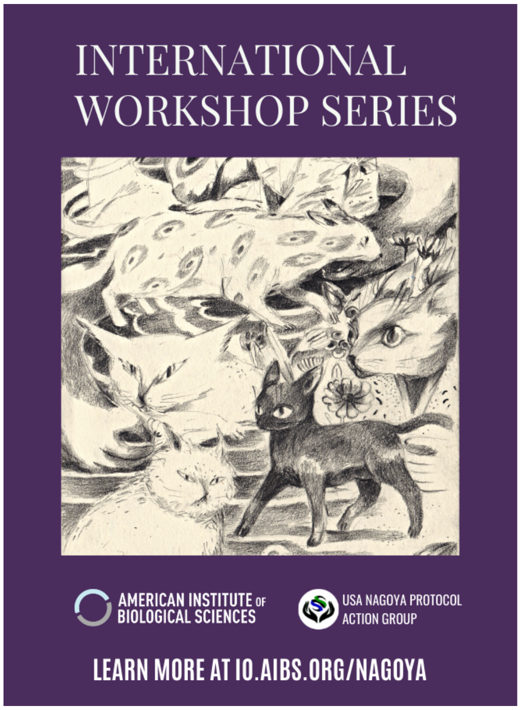

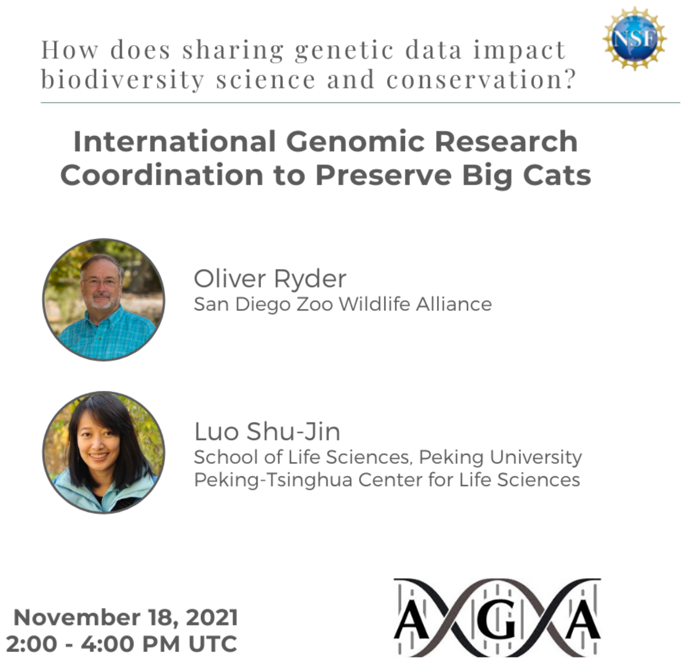

This workshop, hosted by the American Genetic Association, focused on genomic research on big cats across the world, their genetic diversity and challenges in their conservation efforts. It was attended by 92 participants from 27 countries representing North America, South America, Asia, Europe, Africa, and Oceania. The workshop began with an introduction by Dr. Rebecca Adler Miserendino, who discussed the definition of DSI, various related terminologies, the scope of their usage in different contexts, and issues around DSI sharing.

The first talk "International Genomic Research Coordination to Preserve Big Cats" was presented by Dr. Oliver A. Ryder, director of conservation genetics at the San Diego Zoo Wildlife Alliance, whose research interests include extinction risk, biodiversity and species recovery using molecular genetics, genomic studies and genetic rescue. Dr. Ryder is an advocate for a global network of cryo-banking facilities with tissue culture cells and is also actively involved in steering committees for vertebrate genomics projects. With extensive experience in banking samples and using them for biodiversity genomics, Dr. Ryder shared information about consortia for various taxonomic groups, such as Genome 10K, Vertebrate

Genome, Earth Biogenome and Zoonomia projects. The San Diego Zoo Wildlife Alliance also serves as a biodiversity bank which has been collecting biomaterial samples such as fibroblast cell cultures, gametes and frozen tissue for 40 years.

The second talk "International Genomic Research Coordination to Preserve Big Cats: My Personal Story 2000-2021" was presented by Dr. Luo Shu-Jin, Professor in the School of Life Sciences, Peking-Tsinghua Center for Life Sciences at Peking University, China. It described her work toward studying wildcat genomic diversity, evolution and conservation over a twenty-year career. Her laboratory in China focuses on the genomic diversity, evolution and conservation of wildcats, particularly tigers. Captive tiger populations (numbering up to 20,000) have been bred in captivity over 200 years, but only a small number of captive tigers are in well-maintained conservation breeding programs, while the rest are termed as 'generic tigers', with their origins untraceable and being bred for entertainment purposes. This has made studies on tiger evolution logistically difficult. The 4000 wild tigers remaining range over 13 countries, creating challenges with different national regulations.

Dr. Luo described the Washington Convention, also called CITES (Convention on International Trade in Endangered Species), which regulates the use of biological samples, including specimens, tissue samples, and DNA but excludes in-vitro products generated from PCR reactions. The study of CITES-protected species necessitates establishing a local collaboration in the source country prior to sampling and applying for export permits, quarantine procedures, import permit for the user and actualizing transport, shipment and passing customs declaration of the material. CITES registered scientific institutions can use a Certificate of Scientific Exchange (COSE) to allow loans of accessioned samples. While greater regulation of DSI would add barriers to innovation, open access to data will maximize value to the scientists and society in both the user and provider countries as the distinction between the two continues to blur.

Discussion

The discussion session started with questions about the relationship between wildlife genetic data and the broader aspects of climate or landscape change and using genetic data in this regard. Defining responsible data use was discussed, followed by brainstorming for best practices. While the importance of open data sharing was acknowledged, large databases often generate economically valuable products that are not clearly governed by legislation. Block-chain technologies could be a possible way forward, but the high computational power costs and organizational issues could render them ineffective. Best practices could also involve acknowledging collaborators, determining data entry protocols, tracking origins of the genetic data, complying with the Nagoya protocol while publishing and self-policing by researchers. Other measures could include volunteering with working groups to help connect data stored on various repositories such as GBIF and NCBI and setting up frameworks that are sensitive to certain data.

Recommendations

The following ideas emerged from conversations taking place during the workshop:

1. Accelerate generation of affordable genomic data in the provider country or a neutral genomics core in order to simplify permit processes, through in-situ sequencing and remote data sharing.
2. Apply lessons from the Human Genome Project, the Bermuda Principles and the Fort Lauderdale Agreement that advocate for free use of genomic data by the scientific community before publication purposes.
3. Support the autonomy of international long standing programs to create and maintain their own bioethical and data access standards. Programs are effective vehicles to deliver benefits and measure accountability.
4. Researchers should prioritize acknowledging collaborators, determining data protocols, tracking origins of the genetic data, complying with the Nagoya protocol while publishing and self-policing by researchers.
5. Encourage researchers to volunteer with working groups to help connect data stored on various repositories, such as the Global Biodiversity Information Facility (GBIF) and the National Center for Biotechnology Information (NCBI) (e.g., GenBank).

Workshop VI: Digging into the Bioethics of Studying Humans, from Culture to the Genome
([Watch Recording](#))
Topic: Anthropology, Ethnobiology and Paleobiology

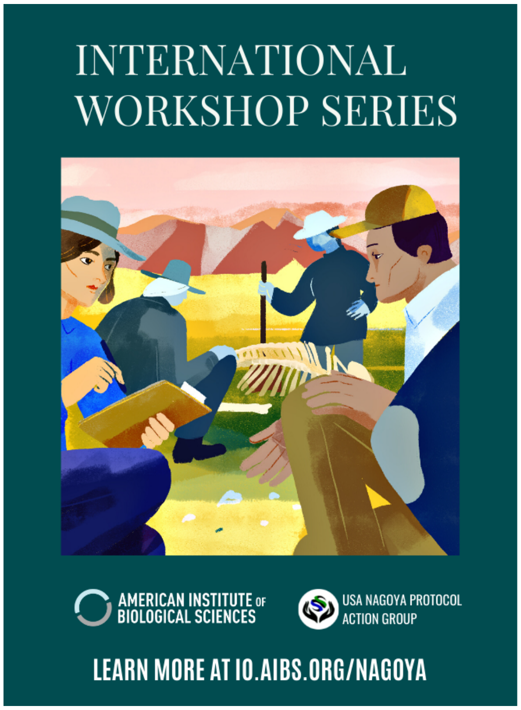

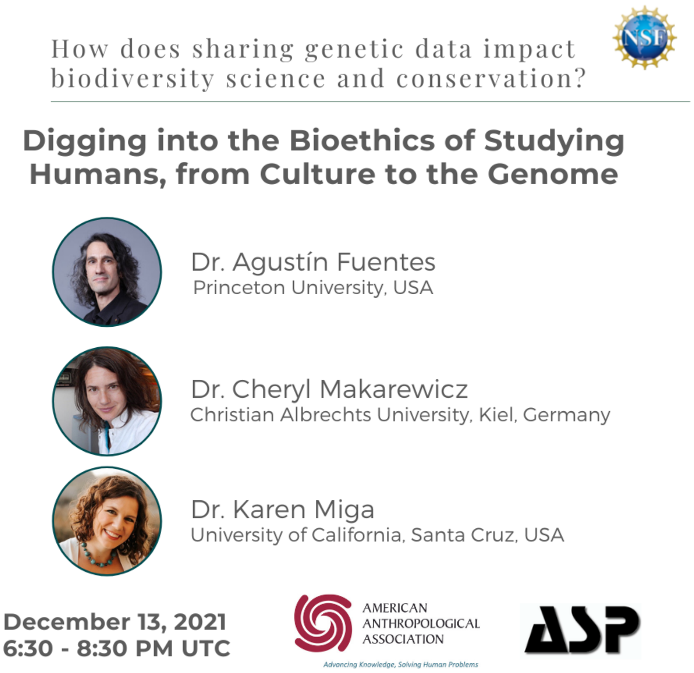

The final workshop, hosted by the American Anthropological Association and the American Society of Primatologists, focused on the bioethics of studying cultural and genomic aspects of

humans. It was attended by 39 participants representing North America, South America, Asia, Europe and Africa. The workshop began with an introduction by Dr. Rebecca Adler Miserendino, who discussed the definition of DSI, various related terminologies, the scope of their usage in different contexts, and issues around DSI sharing.

Dr. Agustín Fuentes, Professor of Anthropology at Princeton University, USA, presented a talk, titled "Rethinking the Center/Periphery: Inequity, Collaboration and Benefit sharing for DSI in Human and other-than Human Primate Studies." Dr. Agustín Fuentes, Professor of Anthropology at Princeton University, focuses on the biosocial, delving into the entanglement of biological systems with the social and cultural lives of humans, our ancestors, and a few of the other animals with whom humanity shares close relations. He shared lessons learned from research projects including studying human-macaque interface in the long-tailed macaque species and exploring the relationship between DNA, bodies and behavior in humans. One ambitious international collaborative project had to be halted because of increasing conflicts about the intended usage of DSI, interpretation and dissemination of results, and associated legal structures among local, national (Indonesian) and international collaborators. Another large-scale project on human ancient DNA (aDNA) funded by the National Geographic Foundation, the John Templeton Foundation and coordinated by prominent aDNA researchers resulted in informal agreements and white papers around issues in human genetic research, but also led to carving an unequal space where certain research groups took center stage whereas the concerns of smaller collaborators were largely excluded from deliberations on pervasive aDNA-research related conflicts.

The next talk was presented by Dr. Cheryl Makarewicz, Professor of Zooarchaeology and Stable Isotope Biogeochemistry at Kiel University, Germany: "Ancient Source Specimens and DSI: Access and Benefits-sharing for our Collective Human Past." Dr. Makarewicz introduced the broad theme of her research on the relationship between shifts in human-animal relationship and their role in shaping subsistence, society and symbolic systems and discussed how archeological datasets can inform current challenges, especially as ancient livestock genomics involves DSI. While genomics is crucial to address various questions, it is heavily supported by zooarchaeology, stable isotopes and proteomics to integrate different kinds of information on demography, production goals, ritual behaviors, scales of animal mobility, dietary intake, taxonomic resolution and diseases. Dr. Makarewicz presented two projects on human-animal relationships, namely the Near Eastern animal domestication project which studies initial human experimentation with animal husbandry, single versus dispersed domestication events, and subsistence innovations, and the ASIAPAST Project, which traces translocation pathways of domesticates across the Eurasian steppe, population histories, and selection for specific economic traits. Project challenges revolved around hoarding of specimens in the hands of a few dominating research groups and unhealthy competition to secure them, as well as asymmetry between the relatively faster-paced genomic analyses versus slower archeological excavations, and often strong disagreements in the interpretations of results drawn by ancient genomic practitioners and archeologists, particularly when genomic data are over-relied upon and ignore decades over empirically derived archeological data.

The final talk by Dr. Karen Miga, Assistant Professor of Biomolecular Engineering at the University of California at Santa Cruz, USA, was titled "Creating a Reference Genome that Best Serves Humanity: The Promise (and Challenges) of Open Data Sharing." Dr. Miga is the Director of the Human Pangenome Reference Consortium (HPRC) at the University of California, Santa Cruz. This NHGRI-funded initiative is designed to release a human pangenome reference, constructed from more than 350 individuals representing highly diverse ethnic backgrounds, to serve as the foundation for future biomedical and genomic medicine research. After twenty years following the HGP, there is still no specific universal policy that enforces research groups to share their human genomic data or use a particular format or database. Dr. Miga presented various solutions and proposed creating a new international human reference genome, since the current data is inadequate in representing worldwide human genomic diversity which impacts biomedical research, and in turn, propagates inequitable health outcomes. The Global Alliance for Genomics and Health (GA4GH) initiative seeks to bring together a federated team of scientists to improve the reference genome, bioinformatic resources, workflows and create forums for equitable research training outreach opportunities to ensure broad implementation.

Discussions

Speakers acknowledged that the foundation of the Nagoya Protocol was based on inequities between the Global North and the Global South. Furthermore, constantly evolving procedures, colonialism, and heterogeneity interact with legacies and histories of distrust. Discussions revolved around the politics of knowledge, center vs. peripheral issues presented by Dr. Fuentes and what it means to 'decolonize' genomic research. Speakers suggested this would mean directing the objectives, funding and framing of projects away from global north powers towards the peripheral countries that are often at the center of the project. Panelists acknowledged the need for funding bodies to restructure basic funding parameters and center them around enhancing local infrastructure and knowledge. Since many underfunded countries lack the funds to develop research infrastructure, capacity building programs in the form of student training and knowledge transfer would provide important benefit sharing mechanisms.

Speakers highlighted the need for researchers to think about data ownership, control and beneficiaries from any international research. Ideas about creating guides to lead well-meaning intentions into responsible action were also discussed. Such actions include being open with and listening to local indigenous communities, building long-term trust by creating a network of researchers with existing deeper connections with local communities, and encouraging more training. Furthermore, including *in-situ* infrastructural capacity development is needed to have effective multi- or bilateral agreements.

Recommendations

The following ideas emerged from conversations taking place during the workshop:

1. Consider and plan NP regulations that can be the functionality of unstable and complex legislations around archeological sites and objects that makes regulation by modern geo-political identities impractical or difficult. Claims and national sentiments

surrounding the ancient specimens should be taken into account with special focus on unresolved political landscapes.
2. Bring the objectives, funding and framing of projects closer to the source from global north powers towards the peripheral countries that are often at the center of the project.
3. Publish in languages other than English to promote equity and information sharing outside Euro-American scientific circles.
4. Consider using federated data sharing platforms that appropriately handle sensitive data and ABS agreements.
5. Build long-term trust by creating a network of researchers with existing deeper connections with local communities.
6. Provide more support for student training and knowledge transfer.
7. Funding bodies should consider restructuring funding parameters to incorporate mechanisms to enhance local infrastructure and knowledge.
8. Increase *in-situ* infrastructural capacity.

5. Overall Recommendations

During the series, it was widely recognized that benefits arising from the use of genetic resources, including data and knowledge, should be shared in a fair and equitable way. But participants also expressed concerns that restrictive regulation of DSI or limits on the open sharing of DSI could hinder scientific progress, biodiversity conservation, and innovation.

Several broad recommendations emerged from the workshops that can contribute to the upcoming discussions on DSI. These are summarized below.

1. Negotiators need to continue exploring policy options that differentiate between commercial and non-commercial use of DSI, which can be practically implemented, and enable the open sharing of DSI across international borders without delay.
2. Negotiators should establish an open forum for major public DSI databases, data aggregators, and users of DSI to explore mechanisms to improve tracking of provenance data and linkages among DSI, traditional knowledge, and specimens.
3. Negotiators should establish a forum under the auspices of the Convention on Biological Diversity to host international scientists and other users of DSI to develop international best practices for cross-border collaboration.
4. Negotiators should:
    a. Urge parties to engage with their domestic science funding agencies to provide institutional-level support to develop domestic infrastructure to support NP compliance.
    b. Encourage non-parties to voluntarily follow the guidance in (4.a.), recognizing that scientists from non-party countries must also comply with the legal frameworks established by NP parties to conduct international research and data sharing.

5. The CBD Secretariat should develop outreach materials to ensure that institutions and scientists understand their obligations pertaining to the access and benefit sharing of physical specimens and to DSI, as appropriate.
6. Recognizing the exemplary practices of growing ABS around DSI in the collections community, negotiators should:
    a. Direct parties to increase resources to support the development, operations and maintenance of biorepositories.
    b. Invite non-parties to voluntarily contribute to these efforts.
7. Parties and non-parties should be encouraged to enhance support of bilateral and multilateral capacity building, collaboration, training and educational exchange programs.
8. Negotiators should encourage states not party to the Convention on Biological Diversity to ratify the agreement as soon as practicable.

These recommendations are also captured in a forthcoming Viewpoint article published in the journal *BioScience* (Adler Miserendino et al., 2022).

6. Conclusion

As policy options are considered for how to address DSI in the context of existing ABS mechanisms, including the NP, the implementation framework for each option must be reviewed to assess potential impacts on future technological developments and biodiversity conservation. **Policies must preserve open access to DSI for non-commercial intended research**, **enable international collaboration, be practical, efficient and cost-effective to implement, ensure legal certainty, and account for both monetary and non-monetary benefits;** see Scholz et al. (2022) for one recently proposed multilateral mechanism that decouples DSI access from benefit-sharing and addresses these concerns. The scientific community recognizes that the responsible, open availability of DSI democratizes biological research, biodiversity conservation, and innovation. As highlighted in this international workshop series, the scientific community continues to improve its practices to broaden capacity and enhance intellectual collaboration as a part of benefit sharing with international counterparts. To optimize the effectiveness of ABS frameworks for DSI, policy makers and negotiators must create opportunities to engage with research stakeholders, including DSI users, public biorepositories, data aggregators, science funding agencies, and international scientific societies and enhance support for international biodiversity-focused research and NP compliance.

Important Links

1. Workshop Series Recordings Playlist on YouTube:
   a. Emerging Pathogen Research Across the Americas: https://www.youtube.com/watch?v=ivKfaohRHq8
   b. The Role of International Collaborations in Resolving Viral Diseases of Cassava in Africa: https://www.youtube.com/watch?v=BB58bZjXRKA
   c. Enabling Large-Scale Ecological Research with Open Genetic Data: https://www.youtube.com/watch?v=S7tfLmeSiCY
   d. More than Potatoes: Collaboration for Collecting and Building the Tree of Life: https://www.youtube.com/watch?v=1_3xoq8LCTg
   e. International Genomic Research Coordination to Preserve Big Cats: https://www.youtube.com/watch?v=jGFvkABqDU8
   f. Digging into the Bioethics of Studying Humans, from Culture to the Genome: https://www.youtube.com/watch?v=GgphR7h84S4

2. Workshop Series announcement on AIBS website: io.aibs.org/nagoya

3. Workshop Series Landing Page on the Learn Nagoya Learning Portal (LearnNagoya.com): https://learnnagoya.com/workshop-series/

4. Individual Workshop Pages:
   a. Emerging Pathogen Research Across the Americas: https://learnnagoya.com/aeid-workshop/
   b. The Role of International Collaborations in Resolving Viral Diseases of Cassava in Africa: https://learnnagoya.com/crops-workshop/
   c. Enabling Large-Scale Ecological Research with Open Genetic Data: https://learnnagoya.com/macrosystems_lter/
   d. More than Potatoes: Collaboration for Collecting and Building the Tree of Life: https://learnnagoya.com/phylogenetics-workshop/
   e. International Genomic Research Coordination to Preserve Big Cats: https://learnnagoya.com/vertebrates/
   f. Digging into the Bioethics of Studying Humans, from Culture to the Genome: https://learnnagoya.com/digging-into-the-bioethics-of-studying-humans-from-culture-to-the-genome/

References Cited

Adler Miserendino, R.A., Meyer, R.S., Zimkus, B.M., Bates, J., Silvestri, L., Taylor, C., Blumenfield, T., Srigyan, M., Pandey, J.L., 2022. The case for community self-governance on access and benefit sharing of digital sequence information. *BioScience* (in press).

Bakelana, Z., Laura, M.B., Mahungu, N., Mavila, N., Matondo, M., Nlandu, N., ... & Kanana, T. (2019). First report and preliminary evaluation of cassava root necrosis in Angola.

International Journal of Agriculture, Environment and Bioresearch. 4(3), 2019. 37-46.

Colella JP, Bates J, Burneo SF, Camacho MA, Carrion Bonilla C, Constable I, et al. (2021). Leveraging natural history biorepositories as a global, decentralized, pathogen surveillance network. *PLoS Pathogens* 17(6): e1009583. https://doi.org/10.1371/journal.ppat.1009583

Convention on Biological Diversity (CBD) Decision 14/20, Decision Adopted by the Conference of the Parties to the Convention on Biological Diversity, CBD/COP/DEC/14/20 (November 2018). https://www.cbd.int/doc/decisions/cop-14/cop-14-dec-20-en.pdf

Friso F., Mendive F., Soffiato M., Bombardelli V., Hesketh A., Heinrich M., Menghini L., Politi M. Implementation of Nagoya Protocol on access and benefit-sharing in Peru: Implications for researchers. *J Ethnopharmacol*. 2020 Sep 15;259:112885. doi: 10.1016/j.jep.2020.112885. Epub 2020 Apr 18. PMID: 32311487.

Huddart, J.E.A., Crawford, A.J., Luna-Tapia, A.L., Restrepo, S., Di Palma, F. EBP-Colombia and the bioeconomy: Genomics in the service of biodiversity conservation and sustainable development. *Proceedings of the National Academy of Sciences*. 2022, 119 (4) e2115641119; DOI: 10.1073/pnas.2115641119

Nagoya Protocol (NP) Decision 3/12, Decision Adopted by the Parties to the Nagoya Protocol on Access and Benefit Sharing, CBD/NP/MOP/DEC/3/12 (November 2018). https://www.cbd.int/doc/decisions/np-mop-03/np-mop-03-dec-12-en.pdf

Scholz, A.H., Freitag, J., Lyal, C.H.C. et al. Multilateral benefit-sharing from digital sequence information will support both science and biodiversity conservation. Nat Commun 13, 1086 (2022). https://doi.org/10.1038/s41467-022-28594-0

Thiers, B., Bates, J., Bentley, A.C., Ford, L.S., Jennings, D., Monfils, A.K., Zaspel, J.M., Collins, J.P., Hazbón, M.H. and Pandey, J.L., 2021. Implementing a Community Vision for the Future of Biodiversity Collections. *BioScience*, 71(6), pp.561-563.

Wilkinson, M. D. et al. (2016) The FAIR Guiding Principles for scientific data management and stewardship. *Sci. Data* 3:160018. doi: 10.1038/sdata.2016.18. https://www.ncbi.nlm.nih.gov/pmc/articles/PMC4792175/

Glossary

ABS: Access and Benefit Sharing
CARE: Collective Benefit, Authority to Control, Responsibility, Ethics
CBD: Convention on Biological Diversity
CITES: Convention on International Trade in Endangered Species of Wild Fauna and Flora
DSI: Digital Sequence Information
DiSSCo: Distributed System of Scientific Collections
FAIR: Findable, Accessible, Interoperable, Reusable data
GBIF: Global Biodiversity Information Facility
NCBI: National Center for Biotechnology Information (e.g., GenBank)
NP: Nagoya Protocol on Access to Genetic Resources and the Fair and Equitable Sharing of Benefits Arising from their Utilization

Workshop Steering Committee

The workshop series was organized by a steering committee, co-chaired by two of its members as described below:

- **Jyotsna L. Pandey (PI)** is the Director of Public Policy for the American Institute of Biological Sciences in Herndon, VA and Executive Director of the Natural Science Collections Alliance.
- **Rachel Meyer (co-Chair of Steering Committee, co-PI)** is an assistant adjunct professor in Ecology and Evolutionary Biology at University California Santa Cruz.
- **Rebecca Adler Miserendino (co-Chair of Steering Committee)** is a Principal and the Head of International Practice at Lewis-Burke Associates in Washington, DC.
- **John Bates (co-PI)** is curator of birds at the Field Museum in Chicago, Illinois and President of the Natural Science Collections Alliance.
- **Crispin Taylor** is CEO at the American Society of Plant Biologists in Rockville, Maryland.
- **Breda Zimkus** is the Assistant Director of Collections Operations at the Museum of Comparative Zoology, Harvard University.

Authors of the Report

In addition to the workshop steering committee, the following individuals were commissioned to assist with writing the final report:

- **Tami Blumenfield**, School of Ethnology and Sociology, Yunnan University, People's Republic of China; and Department of Anthropology, University of New Mexico, Albuquerque, New Mexico.
- **Megha Srigyan**, Department of Ecology and Evolutionary Biology, University of California Santa Cruz

This material is based upon work supported by the National Science Foundation under Grant Number 2136950.
